# User-focused data sharing agreements: a foundation for the genomic future

**DOI:** 10.1093/jamiaopen/ooz043

**Published:** 2019-10-01

**Authors:** Carolyn Petersen

**Affiliations:** Division of Biomedical Statistics and Informatics, Global Business Solutions, Mayo Clinic, Rochester, Minnesota, USA

**Keywords:** Information dissemination, data sharing, genomics, genetic testing, tissue donors

## Abstract

Data sharing agreements that clearly describe what individuals are agreeing to and what responsibilities data stewards will undertake are crucial for the establishment, maintenance, and flourishing of genomic datasets. To optimize genomic data resources, researchers, care professionals, and informaticians must regard system design, user objectives, and environmental considerations through users’ eyes, identifying fundamental values on which to build and potential barriers to success that must be avoided. Design of agreements that promote desired data sharing and protect valuable data resources as necessary begins with a review of user interests and concerns. Nontraditional approaches for informed consent (eg, abbreviated informed consent, electronic informed consent, and dynamic consent) can facilitate achievement of data donors’ privacy-related goals while making data available to researchers. Transparency in individual-researcher interactions, recognition and accommodation of cultural differences, and identification of shared needs and goals create a foundation for data sharing agreements that work over short and long terms.

## INTRODUCTION

The ability to sequence human genomes at scale and at reasonable cost has created a pathway to advance science and develop treatments that better match the physiology of the people who will receive them. Initiatives such as the National Institutes of Health’s All of Us Research Program^1^ have the potential to bring the use of genomic information to bear upon the lives of individuals who are not ill and have not undergone genetic testing. When NIH has achieved its goal of collecting the genomic information of one million individuals, it will have set the stage to change the way basic and clinical research are done, potentially facilitating new diagnostic and treatment possibilities.

Presentations of personalized and precision medicine in popular media typically frame them as genetics-based approaches, and have left the public with an optimistic view of what can be accomplished without an accurate accounting of the potential negative effects and the challenges still to be resolved.^2^ For example, the American Society of Human Genetics has emphasized that genetic screening results should be returned to patients with appropriate context,^3^ but a Web-based platform for return of carrier results to adults has performed as well as genetic counselors,^4^ laying the groundwork for results return with little or no human involvement. Genetic testing for hereditary cancer syndromes is accessible to just a fraction of those likely to benefit, but now a digital tool can accurately match such individuals to National Comprehensive Cancer Network testing criteria,^5^ potentially enlarging an already unmet need. Multiple research initiatives already have demonstrated that individuals can be identified via their genomes.^6^^,^^7^ As people gain a deeper understanding about how the interconnectedness of electronic health records, social media, mobile health devices, the Internet of Things, the wide range of publicly available datasets, and other technologies may affect their privacy and impact their lives, they may reasonably experience anxiety about the implications of sharing genomic and genetic information beyond their care team.

Embedded in the healthcare system and the culture are questions of fairness, of balancing achievement of the greatest good for the greatest number with the individual right of autonomy, of providing reasonable recompense to individuals who have provided something of incalculable value, and of how to ensure that products and processes are commercialized in ways that result in equitable access and distribution across the society that provided the resources (eg, tax revenue, research funding, tissue donation, etc.) underlying these advances. Market-focused societies may emphasize monetary remuneration, but other benefits such as information (eg, return of test results),^8^ opportunity for collective community determinism,^9^ and training in research skills may provide meaningful value. Discussion of data sharing agreements must necessarily address questions of equitable distribution of benefits as well as distribution of resources.

In addition, the nature of genomic data gives rise to unique privacy considerations and conundrums. Unlike other data included in electronic health records, genomic data is self-identifying: it cannot be de-identified, and the identity of the individual who produced it cannot be obscured.^10^ When an individual undergoes testing in the course of seeking treatment for a health condition, he or she may receive information that is unrelated to the current concern and perhaps undesired or nonactionable. Genomic and genetic testing may reveal confidential information about not only the person tested but also about family members who may not have sought or welcome this information, creating ethical challenges related to disclosure.^11^

Aside from the technical work still to be done, numerous ethical and social questions remain, among them how to meaningfully describe genomic research so that individuals can provide informed consent, how to frame the challenges associated with protecting privacy and maintaining confidentiality while using genomic datasets, and how to ensure that research results are translated into benefits for all.^12–14^ Although the healthcare system supports stakeholder engagement at the individual level, policy development related to genomic data sharing and management may most effectively be undertaken at the community level.^15^ As innovations accrue and the field advances, finding ways to engage with individuals that feel safe, respectful, and beneficial to individuals as well as the larger society will only become more important.

## INDIVIDUALS’ DECISION-MAKING: N OF 1

Some concerns about data collection and sharing expressed by individuals are universal, transcending culture, language, and community norms.

- *Data security:* Data security and protection of personal information from unintended access and unapproved use is a common theme in investigations of patient and consumer attitudes about data sharing, in both work related to medical information in general and to genetic information.^16–18^ In one study conducted in several countries, patients consistently reported data storage in a secure database, monitoring of data used by other researchers, and inability of researchers to identify individuals based on their data as their primary concerns.^17^
- *Curiosity:* Some individuals are curious enough about themselves or their ancestry to be willing to learn negative or potentially distressing information as part of self-discovery.^19^^,^^20^
- *Attitude of openness.* Others choose to share personal genomic data on social media even though many of these social media users believe that privacy of genetic data will be breached.^21^ Some people are more comfortable sharing anonymized medical details than details about their identity.^22^ Individuals also were more comfortable sharing de-identified data for research (76.2%) rather than identified data for health care purposes (57.3%).^23^
- *Concern about psychological impact of test results:* Individuals vary in their desire to learn potentially life-changing negative information about themselves, particularly when there is significant uncertainty about what the results mean for the future.^19^ A lack of treatment for conditions for which an individual is at elevated risk also worries individuals.
- *Broad sharing:* When individuals agree to share their genetic data, a majority want their data made available for as many studies as possible.^24^ Many who have donated tissue samples for genetic research express a desire to benefit the common good, though they may inaccurately distinguish between diagnostic testing and research.^25^^,^^26^ However, providing individuals with greater detail about the risks of sharing data can reduce the number willing to share data and the degree of sharing permitted.^27^^,^^28^

Other concerns that play a role in data sharing decisions vary by individuals’ personal circumstances.

- *Culture:* Culture plays a significant role in attitudes about data sharing.^18^^,^^29^ In a study assessing factors that influenced decisions about data sharing among people with diabetes in Denmark, The Netherlands, Sweden, and the United Kingdom, Danes cared most about controlling the types of data shared while Dutch patients were most concerned with whom data would be shared.^30^ In addition, residents of rural areas report lower awareness of genetic testing.^31^
- *Trustworthiness:* Even when individuals support data sharing in concept, they make distinctions among the organizations with which they will share information, expressing greater reluctance to share information with for-profit organizations.^32^ Individuals also have reported greater willingness to share data with academic researchers than with researchers affiliated with government or industry.^33^
- *Age:* Older individuals may be more comfortable sharing their data, perhaps because they perceive fewer negative consequences should breaches of confidentiality occur.^26^^,^^32^ Older people also express willingness to share data if it is protected.^34^ Parents making decisions about sharing their children’s data prefer more restrictive agreements due to concerns about future risks.^35^ More than a decade after routine newborn screening, parents express concerns about expansion of screening programs.^36^
- *Race:* Awareness of genetic testing to predict the risk of developing cancer has been reported to be lower among ethnic minority groups (eg, African-Americans, Asian-Americans, and Hispanics), and minority individuals aware of testing view it less positively and/or express increased concerns about testing relative to White individuals.^31^^,^^37–39^ Individuals who self-identified as white were more willing to participate in biobank research than others.^40^ Historical experiences of ethnic minorities also may influence individuals’ perceptions of the health care system and willingness to engage in genetic testing.^37^^,^^41^^,^^42^
- *Education:* Individuals with higher educational attainment were more willing to participate in biobank research.^40^ Individuals of ethnic minorities may be more willing to engage in genomic testing with higher levels of education and health literacy.^41^
- *Fewer information needs:* Individuals who need less information about biobank governance and how their information could be used in research studies in order to be able to decide whether to donate sample tissue to a biobank are more likely to be willing to participate.^40^
- *Perception of personal benefit:* Up to 70% of individuals reported an expectation of personal benefit from sharing their data such as better treatment, even when the nature of research studies were not known at the time of consent to participation.^43^ Others expected that data sharing would lead to better patient-provider communication, a greater understanding of their condition, and more personalized treatment.^44^ Yet others agreed to share data as a way to gain access to their personal information.^20^

Although these factors are important, individuals’ stated preferences about data sharing aren’t always borne out by their actions; reported preferences for sharing have proven more restrictive than the choices people make in real-life situations.^45^ This disconnect occurs because risks are viewed as things that happen in the future, whereas benefits will occur more immediately.

## THE RESEARCH VIEW

Just as individuals’ personal circumstances influence their views of genomic sequencing and genetic testing, researchers’ views of genetic testing are influenced by their role(s) within health systems and their relationship(s) with patients and other individuals. This bias plays out in ways that may be at odds with individual patients’ goals, preferences, and needs related to genomics.

Perceptions of clinical roles and responsibilities, the short-term relevance of and time needed to manage at scale treatment-focused genetic testing in newly diagnosed breast cancer patients, and the potential effect on patient care and outcomes were primary considerations among providers with and without specific training in genetic testing.^46^ The care opportunities provided to patients within a particular health system make a difference. Receiving genetic counseling in conjunction with genetic testing empowers patients.^47^ The need for genetic risk information, such as when contemplating testing for the BRCA mutation after breast cancer diagnosis, has been shown to predict information seeking behavior.^48^

Lack of understanding laws and regulations that govern data sharing hamper some researchers.^49^ Though most people have general awareness related to the Health Insurance Portability and Accountability Act and the Genetic Information Nondiscrimination Act, regulations related to biobank operation may be less familiar, particularly when researchers take part in international initiatives. Re-identification of protected health information may not spark concern among researchers because such data can support efforts that cannot be accomplished with de-identified data. However, data re-identification poses a risk for researchers because it can lead potential data donors to distrust researchers and become less willing to participate.^20^

Data governance and management may become even more complex when citizens seek to participate in cross-border initiatives. Although the General Data Protection Regulation (GDPR) applies to all European Union (EU) members, the GDPR does not resolve differences in laws of member countries.^50^^,^^51^ Researchers in EU countries have had opportunities to access transnational large prospective cohorts established by other EU member nations, but legal and regulatory issues (eg, consent) associated with sample and data transfer to non-EU countries remain unresolved.^52^^,^^53^ The need to protect the rights and interests of local data donors and researchers may be at odds with the interests of researchers and institutions elsewhere, and institutional review boards must favor local interests to recruit donors and comply with federal regulations.^54^ Researchers also express concern about proper acknowledgment of data contributions and involvement in governance of all researchers, particularly those from low- and middle-income countries.^54^ Whether managers of biobanks and other data resources would welcome involvement in data governance by individuals who provided data (eg, citizen scientists, patient-powered research networks) remains undetermined. Aligning international policies regarding data sharing and governance, perhaps through an international Code of Conduct,^55^ could facilitate sharing of genomic and health data,^56^ though it is unclear how easily such an agreement could be reached, given the variation in needs, goals, and regulatory restrictions of researchers and institutions worldwide.

Researchers who received a data-sharing agreement have been more willing to share their dataset of individual patient data compared with control participants.^57^ If data donors have not discussed how personal information is to be shared, they may be caught unawares, and may regard the research experience negatively. At the same time, talking about data use and data sharing may be a positive experience for researchers, but not all individuals desire this degree of involvement.

## WORKS IN PROGRESS

The challenges inherent in developing data sharing arrangements that can accommodate researcher and research program objectives, a broad range of data donor needs and goals, legal and regulatory compliance activities, and associated requirements are abundant. Although many considerations have become well understood through decades in the pregenomic era, other issues are just starting to be identified. Though the development of new approaches to informed consent processes is only one of several necessary changes in research process,^58^ it represents a starting point for change. Fortunately, this work is already underway.

### Abbreviated informed consent

The Clinical Genome Resource (ClinGen) has developed an abbreviated consent process to document patients’ agreement to share individual-level data such as genotype and phenotypic elements, diagnosis, and demographic information. Funded by the National Institutes of Health, ClinGen is a collaborative effort to build publicly available genomic databases to support clinical care.^59^ A majority of the 4613 respondents to a Web-based survey indicated support for a consent process involving a one-page consent form and supplemental video.^60^ This work may offer a way to facilitate collection of genomic data obtained outside research initiatives—data that are not subject to the NIH’s Genomic Data Sharing Policy,^61^ which requires researchers to obtain broad consent for sharing of de-identified genomic data.

### Electronic informed consent

Tissue samples that have been stored in biobanks can be a valuable resource for research into genetic and/or rare diseases, particularly when such samples are linked to electronic health records. However, samples cannot be used without the consent of those who provided them. A multimedia, Web-based version of the consent form used by the Partners HealthCare Biobank in face-to-face consent processes offers a less resource-intensive method for seeking consent. During the initial 20-month period, 30% of those invited to participate in the biobank using an electronic informed consent (eIC) tool agreed to do so, while 51% of those invited in person agreed.^62^ Although the acceptance rate was lower for the eIC than for in-person consent processing, the ability to invite patients to participate via a series of email invitations offers an opportunity to reach greater numbers of patients within the same period, potentially resulting in a larger number of participants.

### Dynamic consent

Dynamic consent has been described as an approach for engaging with people about the use of their personal data using an interface that permits individuals to provide and amend consent over time.^63^ This approach allows data donors to give varying types of consent depending on the research involved, agree to data reuse with knowledge of what they are agreeing to, and track all consent decisions in one place. Individuals can opt into specific types of research in one action, or require separate notification and consent for each use of their data. At the same time, research organizations can customize the interface to make data donor communication and consent management efficient, transparent to investigators, and compliant with regulatory and funder requirements. Most important, initial testing indicates that patients regard dynamic consent positively and its use may improve trust and increase engagement in research.^64^

## COMING TOGETHER, DOING BETTER

Individuals, researchers, and others involved in health care have different interests and objectives with regard to secondary uses of genomic information. Data sharing agreements that truly inform data donors at the time of consent and facilitate transparency throughout the data collection, storage, and sharing processes could significantly boost genomic research opportunities. When researchers can accurately and meaningfully frame their proposed activities for individuals, they give individuals the knowledge to distinguish among options and the confidence to commit to a degree of data sharing. Trust is a key issue in transparency, and approaches to data management must be designed to allow individuals to build trust with the holders of their data over time.

Individuals and researchers operate within different cultures that are based on different assumptions, expectations, and goals. Culture is an important factor in how individuals think about data sharing, and the design of data sharing frameworks must accommodate variations in culture to ensure that all users can locate a recognizable point of entry. A shared starting point that functions as a foundation for the data sharing agreement will position all parties for a successful partnership in the immediate future and over time.

## AUTHOR CONTRIBUTIONS

C.P. conceived the idea for this article and wrote and revised the manuscript.

## ETHICS STATEMENT

No human research was undertaken in the course of this work, so IRB approval was neither needed nor sought.

## CONFLICT OF INTEREST STATEMENT

None declared.
